# Determinants of the Financial Contribution to the NHS: The Case of the COVID-19 Emergency in Italy

**DOI:** 10.3389/fpsyg.2020.584473

**Published:** 2020-11-13

**Authors:** Cinzia Castiglioni, Edoardo Lozza

**Affiliations:** Department of Psychology, Catholic University of the Sacred Heart, Milan, Italy

**Keywords:** COVID-19, tax behavior, charitable giving, sustainable development, framing effect, common good, financial contribution

## Abstract

The COVID-19 pandemic has quickly become an unprecedented challenge for many countries at a global level, requiring a significant amount of financial resources to support the National Healthcare System (NHS). In Italy, most of these resources came from the general public through tax payments and monetary donations. The present work aims to investigate the antecedents of citizens’ willingness to financially support the NHS in a situation of public emergency such as the one related to the COVID-19 outbreak. It also aims to distinguish between the willingness to support the system through two different forms of financial contribution, tax payment and charitable giving. An empirical study was performed in the midst of the Italian public health emergency, while the country was reaching its contagion peak. Results showed that participants were more willing to give a financial contribution when it was framed as a one-off donation rather than as a one-off tax payment. Moreover, it was found that trust in money management was the most important factor in predicting the intention to make a financial contribution to the NHS, either through a tax payment or through charitable giving. The perceived risks with regard to the pandemic, in contrast, had no impact.

## Introduction

In late December 2019, an outbreak of the novel coronavirus disease (COVID-19) caused by severe acute respiratory syndrome coronavirus 2 (SARS-CoV-2) began in Wuhan, China, and quickly reached the other countries of the world (Xu et al., 2020). On January 30th, 2020, the World Health Organization (WHO) declared the coronavirus outbreak a public health emergency of international concern, as such influenza pandemics can lead to significant mortality and widespread socio-economic disruption (Bootsma and Ferguson, 2007; Smith et al., 2009). Significant financial resources are critical to respond efficiently to such an emergency, and, historically, most of these resources came from the general public through tax payment and monetary donations. Paying taxes and giving money to charitable institutions, indeed, can be considered two complementary ways of creating public value and increase social welfare (Sugden, 1984; Slavov, 2014). In many countries, tax money provides essential services, including healthcare, whereas charitable giving can alleviate those problems related to the crisis of the welfare state (Selle, 1993). In recent years, issues such as the global financial crisis, growing social inequalities, and the coronavirus outbreak have placed economic sustainable development in the spotlight and increased tension between providing for the common good and focusing on one’s own self-interest. Arguably, for the well-being of our societies, it is crucial to find mechanisms to promote prosocial choices over egoistic ones, including tax compliance and charitable giving. Promoting this kind of prosocial behavior is of paramount importance to meet the UN Sustainable Development Goals (United Nations, 2018). Among different approaches that can shed light on understanding how to reach such sustainable development goals, opening the black box of psychological processes is one of the newest and most promising frontiers (Di Fabio and Rosen, 2018).

Based on these premises, the present work aims to adopt a psychological framework to investigate the antecedents of citizens’ willingness to financially support the national healthcare system in a situation of public emergency such as the one related to the new coronavirus outbreak. It also aims to distinguish the willingness to support the system through two different forms of financial contribution, tax payment and charitable giving. To do so, an empirical study was performed in Italy – one of the first and most affected countries – while it was reaching its contagion peak. The article will be structured as follows: first, an overview of the Italian context (COVID-19 health emergency and financial situation) will be provided; next, a theoretical background to test a model will be presented by adopting the theory of planned behavior (Ajzen, 1991); and finally, the adopted methodology will be described and the main results will be presented. The attitudinal component, normative component, and perceived behavioral control will be examined in relation to the intention to make a financial contribution to the National Healthcare System (NHS) to support the COVID-19 health emergency. Besides the interest at the theoretical level, understanding the antecedents of such financial contribution is of paramount importance to orient public policies and social campaigns.

### COVID-19 Emergency in Italy

On February 21st, 2020, the Italian region of Lombardy became the center of Italy’s coronavirus outbreak when the first locally transmitted case was confirmed. Beginning March 8th, 2020, the Italian government adopted very urgent and restrictive measures to lessen the spread of the virus and its potential impact on the population. Among the more far-reaching measures, any movement of physical persons (both within and entering/exiting the national territory) has been forbidden, except for proven work needs, situations of necessity or health reasons. Schools and universities have been closed, and there are have also been restrictions on the exercise of public activities (sports, restaurants, entertainment, etc.) and any form of gathering of people in public places. In parallel, measures were taken to strengthen the National Health System, with particular reference to intensive care.

Despite such measures, healthcare providers in Italy (and elsewhere) have been called to work in very critical conditions. It was not expected that the infection would spread so rapidly throughout the population and that there would have been such a significant number of serious cases that would require intensive treatment. As a result, the need for beds in intensive care units (ICUs) rapidly exceeded the number of available beds (Nicoli and Gasparetto, 2020). At the time of this writing, more than 240,000 people in Italy have tested positive for the virus and more than 35,000 people have died^1^. It did not take long before the Italian government realized that extraordinary financial measures were needed to support the National Healthcare System and face the pandemic.

Like some other countries, the Italian healthcare system provides universal coverage to citizens and residents, with public healthcare largely free of charge. From an organizational point of view, the system is regionally based, with local authorities responsible for the organization and delivery of health services, leaving the Italian Government with a weak strategic leadership. Over the period of 2010–2019, the National Healthcare System suffered financial cuts of more than €37 billion and a progressive privatization of health-care services. Public health expenditure as a proportion of gross domestic product was 6.6% for the years 2018–2020 and is forecast to fall to 6.4% in 2022 (Cartabellotta et al., 2019). The on-going coronavirus pandemic, together with the already fragile Italian financial situation and the public healthcare expenditure cuts over the years, poses additional challenges in relation to citizens’ health-related rights. For this reason, understanding how to promote and sustain citizens’ financial contributions for the common good–either through tax payments or charitable giving–is of paramount importance.

### Italian Financial Situation

The Italian economy was already in serious difficulty before the coronavirus outbreak, and Italy’s fragile public finances make the issue of financial provision for the common good even more relevant. According to Eurostat^2^, in 2019 the Italian government debt equaled 134.8% of the country’s economic output. In the European Union, where the average for 27 countries is 77.8%, Italy has the second highest ratio of government debt to GDP.

In addition, Italy is a low-tax-compliance country. Although estimates of the level of tax evasion are, inevitably, uncertain, the publicly available data and research carried out over time agree that the phenomenon is significant. According to recent estimates (Schneider, 2015), Italy’s shadow economy is higher (20.6% of GDP) than the average of 31 other European countries (18.0%), and tax evasion is estimated to be much higher than in other highly developed countries (Giovannini, 2011). According to the latest report of the Italian Ministry of Economy and Finance (MEF), the difference between the amount of tax and social contribution that is due and what is actually paid was €109 billion per year, on average, during the period of 2012–2016 (i.e., 6.4% of GDP). Tax evasion is often considered the root of many problems within the Italian economy, such as revenue loss, equity concerns, and other inefficiencies (Santoro, 2010).

In terms of charitable giving, Italy ranked 33rd out of 128 countries on a 10-year World Giving Index, with 38% of the population donating money (CAF Charities Aid Foundation, 2019). According to a survey on a representative sample of Italian people conducted by BVA Doxa^3^ between March 20th–24th, 2020, 24% of the population – almost 10/12 million Italians – claimed to have already made a donation to support the healthcare and hospital sector since the start of the coronavirus emergency. In addition, 35% said they wanted to donate in the upcoming weeks. Compared to the annual data published by BVA Doxa’s annual research on Italians’ solidarity, these figures represent an increase of about 30% compared to the number of Italians who donate every year to scientific, health and equivalent research purposes, which totaled to 8.3 million in 2019. The fact that many people say they have supported charitable causes related to the virus or are willing to do so is very positive. Nonetheless, the Italian healthcare system cannot face the coronavirus pandemic solely by relying on voluntary monetary donations from private citizens.

Several reasons can be found to explain Italians’ apparent different attitudes toward tax payments and charitable giving. The first and most obvious reason is the distaste for the coercive nature of taxes (as compared to voluntary private giving) and the desire of donors to control or target their donation (Li et al., 2011). In the case of charitable contributions, people can make their own decisions regarding which social programs or causes to support, whereas in the case of taxes, taxpayers seldom have the opportunity to earmark their tax payments for specific causes. Another reason could be the perceived inefficiency of government expenditures on general welfare. Nonetheless, Jones (2017) found that when subjects could voluntarily donate to both government agencies and charitable institutions (thus removing the coercive nature of taxes), they gave significantly less to the government than to charity, even after accounting for the relative effectiveness of the two types of institutional expenditures. This leads to another explanation, that of a deep-rooted “tax aversion” bias, a phenomenon by which people may perceive an additional burden associated with tax payments compared to economically equivalent payments that are labeled differently (Fennell and Fennell, 2003; McCaffery and Baron, 2006). The origin of this aversion can be historically identified (see Ferrari and Randisi, 2011).

Based on such premises, the present study aims to investigate whether labeling financial support to the National Healthcare System as a one-off tax or as a one-off donation can affect the willingness to make such a contribution. Because of the “tax aversion” bias, the following hypothesis is formulated:

H1: People will show greater intentions to give a financial contribution to the NHS if such a contribution is labeled/framed as a donation rather than a one-off tax.

### Antecedents of Financial Contributions to the NHS

As mentioned above, the present study aims to test a model to investigate the antecedents of financial contributions to the NHS during a public emergency. Several antecedents have been identified by adopting the theory of planned behavior (Ajzen, 1991).

First, we took into account people’s attitudes toward the pandemic emergency. In fact, people’s risk perception related to the pandemic has been identified as one of the factors contributing to an increase in public participation in adopting preventive measures (Brug et al., 2009; Poletti et al., 2011). Perceived risk and anxiety about catching the virus can lead people to adopt some protective behaviors such as wearing a mask, isolation (Lau et al., 2007; Barr et al., 2008), and increased hand washing (Lau et al., 2007; Rubin et al., 2009). Perceived severity, perceived risk, and anxiety about catching the virus have also been associated with a reduced use of public transport, avoidance of public places and taking leave of absence from work (Sadique et al., 2007; Goodwin et al., 2009; Rubin et al., 2009). No evidence, however, can be found on the relationship between perceived risk and anxiety about catching the virus and the willingness to financially support the healthcare system. Given the existing literature supporting the relation between perceived risk and adoption of preventive measures, we propose the following hypothesis:

H2: People with negative pandemic attitudes (i.e. high perceived risk) will show greater intentions to give a financial contribution to the NHS.

Second, we considered the role played by subjective norms and values. COVID-19 is challenging our position in the world, because on the one side we realize our connectedness to those around us regardless of how distant they appear to be (e.g., geographic distance), yet on the other side we are becoming deeply aware of our individuality because the illness is a threat to our personal wellbeing (Ahmad et al., 2020). Given the global nature of this pandemic, and the fact that supporting the healthcare system can help not only one’s own family and friends but also distant strangers, we decided to consider people’s orientation toward the common good. The common good entails feelings of responsibility for one’s community and compliance with social rules and order. People’s sense of responsibility for the common good has been identified as one of the core dimensions of the construct called “social cohesion” (Schiefer and van der Noll, 2017). Social cohesion requires a minimum degree of commitment to the community as well as the willingness to subordinate personal (and private) needs to the collective (and public) needs of the social environment. The specific motivation to provide for everybody’s needs and make sure that the common good is accessible to anyone has also been shown to be an antecedent of tax compliance and charitable giving (Castiglioni et al., 2019a). We also decided to take into account the dimension of collectivism. Collectivism emphasizes interdependence, prioritizes collective goals over personal goals, and places more importance on social norms than attitudes (Triandis and Gelfand, 1998). Thus, the following hypotheses has been formulated:

H3: People with high levels of collectivism and common good orientation will show greater intentions to make a financial contribution to the NHS.

Finally, we considered perceived behavioral control. Both tax payments and monetary donations involve money, which is fungible. The effectiveness of one’s financial contribution to the HNS is therefore subsidiary to the actual use and management of that money, which is under the control of a third party (e.g., the government or other institutions/nongovernmental organizations); thus, this evokes trust-related issues (Castiglioni et al., 2018). Understandably, one may be less likely to donate if he or she is concerned that the donation will not reach the impacted people (i.e., low perceived control). Donors want to be assured that their financial contributions will be used for the stated purpose (Oosterhof et al., 2009). Similarly, tax compliance increases when taxpayers are aware of a direct link between their tax payments and the provision of a desirable public good (Alm et al., 1992). Thus, we expect that the level of trust in money management – which is also connected to the general trust people have toward NHS and public institutions–has an influence on the intention to make a financial contribution to the NHS.

H4: People with high levels of trust in money management will show greater intentions to make a financial contribution to the NHS.

**FIGURE 1:**
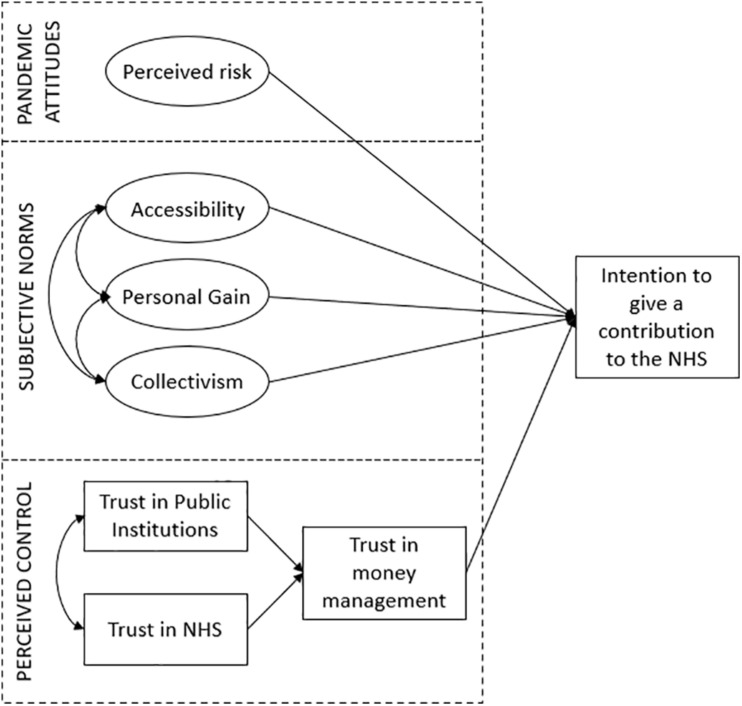
Model of antecedents of the intention to make a financial contribution to the NHS.

In sum, we propose a model of antecedents of financial contributions to the NHS to support the emergency situation that includes attitudinal factors (i.e., pandemic attitudes and perceived risk), subjective norm factors (i.e., orientation toward the common good and collectivism), and perceived control (i.e., trust in money management, public institutions, and NHS). The model is represented graphically in Figure 1. Understanding the relative importance of each factor in determining the financial contribution to the NHS can become a key factor for policy-makers to decide how to develop social marketing campaigns (i.e., in terms of rhetoric to use and key messages to deliver) aimed at increasing financial resources for NHS.

## Materials and Methods

### Sample and Procedure

A between-subjects experimental design was developed through the online survey platform Qualtrics. The questionnaire was preceded by an electronic consent form which contained an adequate disclosure regarding the objectives of the study. Moreover, anonymity was assured to all participants, who were also informed about the possibility to withdraw from the study at any time with no consequences. No incentives were offered for participation. After providing informed consent, participants were automatically randomly assigned to one of the two different conditions (tax group vs. donation group) and read a message in which the financial contribution to the NHS was framed as either a one-off tax or a one-off donation. After reading the message, participants answered a question about their intentions to either pay a one-off tax to support the NHS (tax group) or give money to a charity supporting the NHS (donation group). Further individual difference measures and demographics were collected as detailed below.

The questionnaire was made accessible from March 21st, 2020, to April 8th, 2020. A combination of convenience and targeted sampling was adopted, which included emails to personal contacts and invitations to take part in the study through social media websites (Facebook, LinkedIn, and Instagram).

### Measures

#### Dependent Measures

According to the experimental condition, participants indicated either their intentions to pay an one-off tax to support the NHS (behavioral intentions on a scale from 0 = not at all likely to 10 = extremely likely: “Imagine that in the next few weeks the government decided to place an one-off tax on private citizens to support the National Healthcare System. How inclined would you be to give money to support it?”) or their intentions to make a one-off donation to a charity supporting the NHS (behavioral intentions on a scale from 0 = not at all likely to 10 = extremely likely: “In the last few days, several charities have been collecting money to support the National Healthcare System. How inclined would you be to give money to support it?”).

#### Pandemic Attitudes and Perceived Risk

Five elements related to pandemic risk attitudes were measured: (a) risk susceptibility (i.e., perceived likelihood of contracting COVID-19: participants were asked to rate from 1 (very low) to 5 (very high) their perceived risk of being infected by the COVID-19 virus); (b) risk severity [i.e., perceived potential severity of COVID-19 infection for their own health: participants answered a question regarding how dangerous the effects of COVID-19 could be to their own health, ranging from 1 (not dangerous) to 5 (very dangerous)]; (c) general concern [i.e., personal concerns regarding the COVID-19 emergency at large, ranging from 0 (not at all concerned) to 10 (extremely concerned)]; (d) concerns for their own health and safety [ranging from 0 (not at all concerned) to 10 (extremely concerned)]; and (e) concerns for the health and safety of their loved ones [ranging from 0 (not at all concerned) to 10 (extremely concerned)]. These five elements showed very good scale reliability (α = 0.80).

#### Values and Subjective Norms

Collectivism was assessed using CVSCALE (Yoo et al., 2011), which consisted of six items (e.g., “Group welfare is more important than individual rewards”) evaluated using 5-point Likert-type scales anchored by 1 = “strongly disagree” and 5 = “strongly agree.” The scale showed very good reliability (α = 0.79). Personal orientation toward the common good was assessed using the Common Good Provision scale (CGP, Castiglioni et al., 2019a), which comprises two main dimensions: (a) Accessibility (four items, such as “If I provide for the common good, I do so to provide for everybody’s needs”, α = 0.88); and (b) Personal Gain (three items, such as “If I provide for the common good, I do so to get a personal return,” α = 0.83). Both dimensions were assessed using 9-point Likert-type scales anchored by 1 = “strongly disagree” and 9 = “strongly agree.”

#### Trust and Perceived Control

Participants were asked to express their level of trust toward the National Healthcare System (NHS) and toward public institutions using 5-point Likert-type scales. They were also asked to express their trust in money management; that is, the extent to which they believed the money contributed to supporting the NHS – either through tax payments or donations – would actually be used for that purpose (from 0 = extremely unlikely, to 10 = extremely likely).

#### Sociodemographic Variables

Socio-demographic data were also collected, including age, sex, education, employment situation, region of residency and level of income, in order to characterize our sample.

### Analysis

**FIGURE 2:**
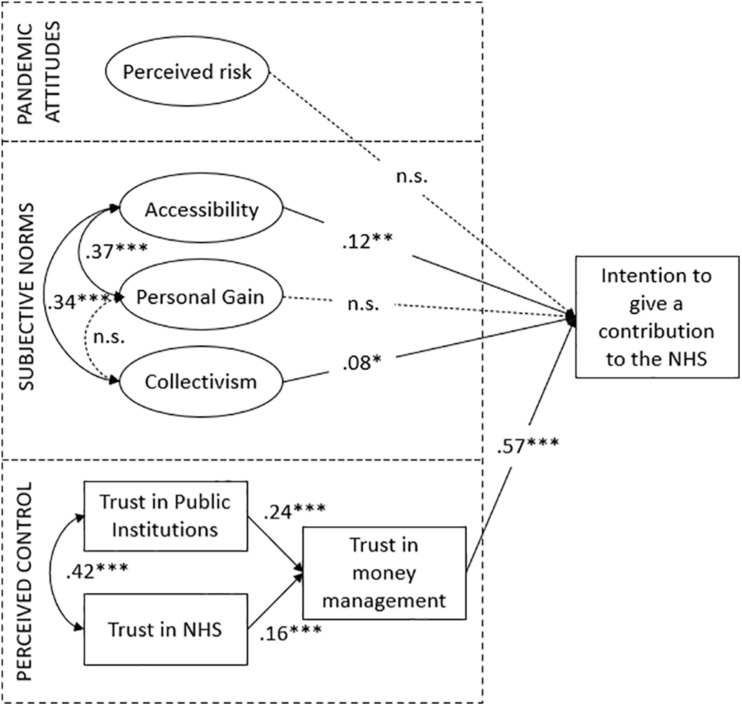
Final model of relationships between antecedents and intention to make a financial contribution to the NHS (*N* = 600). Standardized parameter estimates for the final model. ***p < 0.001; **p < 0.01; *p < 0.05.

**FIGURE 3:**
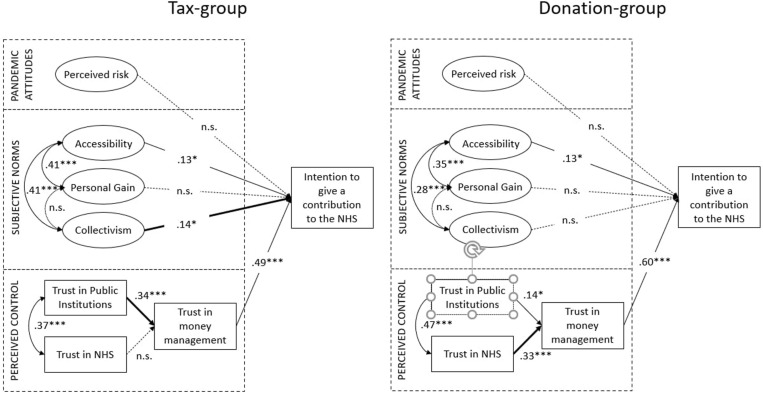
Final model of relationships between antecedents and intention to make a financial contribution to the NHS (Tax group = 298; Donation group = 302). Standardized parameter estimates for the final model. ***p < 0.001; **p < 0.01; *p < 0.05.

Descriptive statistics were computed for each item (asymmetry, kurtosis, mean, median, and standard deviation), and normality of distribution was checked. Cronbach’s alphas were calculated to assess reliability of item scales. The effects of different frames (taxes vs. donations) on the intention to give a financial contribution to the healthcare system was assessed using one-way analysis of variance (ANOVA). Structural Equation Modeling (SEM) was used to examine the model fit. We specified antecedents as exogenous predictor variables and intention to financially provide for the NHS as outcome variable. For greater clarity, neither the regression errors nor the corresponding observable variables are shown in Figures 2, 3. Absolute and relative fit indices are reported in order to determine the fit of the proposed model.

## Results

### Sample Characteristics

We collected 600 valid questionnaires. Participants were Italians (66.5% female) aged between 18 and 75 years (*M* = 33.3, *SD* = 13.8). The majority (57.5%) of participants held a university degree (either bachelor’s or master’s). A high percentage (55.3%) were workers, whereas 35.3% were students and the remaining 10.4% included unemployed people, homemakers, and retired people. The vast majority of respondents came from Lombardy (72%), the region most affected by coronavirus. Regarding their income, 18.8% reported a monthly net household income lower than €1,500, 50.5% reported between €1,500 and €3,500, and 30.7% reported € 3,500 or more.

With regard to the different experimental conditions, 298 (49.7%) comprised the tax payment group and 302 (50.3%) comprised the donation group. Chi-square tests were conducted to test the homogeneity of two different groups for socio-demographic characteristics, and a one-way ANOVA was performed to compare age. No significant differences were found between the tax payment group and the donation group, thus suggesting that random assignment was valid ex post.

### Diagnostics for Normality

Before performing other statistical analysis, descriptive statistics were computed for both dependent and independent variables (asymmetry, kurtosis, mean, median, and standard deviation), and normality of distribution was checked for both groups (tax-group and donation-group). Table 1 shows results of descriptive statistics for each subgroup. All values appear to be in the acceptable range for normal distribution.

**TABLE 1:** Descriptive statistics for dependent and independent variables.

Variable	Group	M	SD	Md	A	K
Intention to make a financial contribution to the NHS	Tax group	5.6	3.1	6	−0.4	−0.9
	Donation group	7.3	2.6	8	−1.0	0.4
Pandemic attitude	Tax group	5.8	1.3	5.8	−0.8	0.7
	Donation group	5.7	1.2	6	−0.6	0.3
Accessibility	Tax group	7.7	1.3	8	−1.3	1.9
	Donation group	7.6	1.5	8	−1.5	1.6
Personal Gain	Tax group	6.7	1.7	7	−0.8	0.4
	Donation group	6.3	2.0	6.7	−0.7	−0.3
Collectivism	Tax group	3.8	0.6	3.8	−0.3	0.7
	Donation group	3.8	0.6	3.8	−0.2	0.3
Trust toward the National Healthcare System (NHS)	Tax group	4.0	0.8	4	−1.1	1.5
	Donation group	3.9	0.7	4	−1.0	1.2
Trust toward public institutions	Tax group	3.1	0.9	3	−0.3	−0.7
	Donation group	3.0	0.9	3	−0.3	−0.5
Trust in money management	Tax group	5.7	2.8	6	−1.2	0.6
	Donation group	7.0	2.6	7	−1.0	−0.7

### Demographic Effects on Intention to Make a Financial Contribution to the NHS

Analysis of variance (ANOVA) was performed to determine the effects of socio-demographic characteristics on the intention to make a financial contribution to the NHS. No significant effects were found for age, sex, education, employment situation, or region of residency. A significant effect was only found for household income [F(2,478) = 7.779, *p* < 0.01, η^2^_*p*_ = 0.04]; thus, income was included as a random factor in the following analysis to determine the presence of framing effects.

### Framing Effects on Intention to Make a Financial Contribution to the NHS

The effects of different frames (taxes vs. donations) on the intention to give a financial contribution to the healthcare system was assessed with one-way analysis of variance (ANOVA). Framing condition was included in the model as a fixed factor, whereas household income was included as a random factor given its association with the dependent variable. Results showed the existence of a statistically significant difference between tax group and donation group [F(1,473) = 43.932, *p* < 0.01, η^2^_*p*_ = 0.95]. Respondents in the tax-framed situation reported a much lower level of intention to make such a contribution (M = 5.6, SD = 2.6) compared to respondents in the donation-framed situation (M = 7.3, SD = 3.1). No significant interaction effect between framing condition and household income was found.

Based on the above evidence, we reject the null hypothesis in favor of the alternative hypothesis suggesting that people show greater intentions to make a financial contribution to the NHS if such contribution is labeled/framed as a one-off donation rather than a one-off tax.

### Structural Equation Model

We tested the model shown in Figure 1 with Structural Equation Modeling (SEM), using the maximum likelihood procedure and the matrix of original data as input with AMOS (Arbuckle, 2003). To test the fit of the model, several indexes are recommended, such as χ^2^ and its level of probability, however, due to its sensitivity to sample size, other fit indexes are proposed, such as the Comparative Fit Index (CFI) and the Root Mean Square Error of Approximation (RMSEA). The model (Figure 2) showed a very good fit to the data: CMIN/DF = 2.030, CFI = 0.94, RMSEA = 0.029 (LO90 = 0.027, HI90 = 0.032). Among the antecedents to the intention to make a financial contribution to the NHS, trust in money management predicted the greatest portion of variance (β = 0.57). As for subjective norms, both the “Collectivism” (β = 0.08) and “Accessibility” (β = 0.12) dimensions had a significant – albeit weaker – positive relation with the dependent variable. For pandemic attitudes and perceived risk, no significant effect was found. Based on this evidence, both H3 and H4 are confirmed. People with high levels of collectivism and common good orientation (in particular, the dimension of “Accessibility” rather than “Personal Gain”) show greater intentions to make a financial contribution to the NHS. Most relevant, the higher is people’s level of trust in money management (which is also determined by the general level of trust toward the NHS and public institutions), the more willing they are to make a contribution to the NHS. No effect was found in relation to perceived risk; thus, H2 is rejected.

After testing the model on the whole sample, we compared the two groups (tax group and donation group) across the same measurement instrument, to see whether path coefficients in the model were equal or different. Figure 3 shows the two structural models, both based on the general model previously tested. Different pathways in the tax and donation framing conditions were found that similarly, explain the intention to make a financial contribution to the NHS.

The first difference in the structural paths between the two groups lies in the antecedent of trust in money management. In the tax-frame condition, trust toward public institutions is the main determinant of trust in money management (β = 0.34) whereas trust in the NHS has no significant impact. This is in line with the fact that, in the tax frame condition, the Italian government is the public institution in charge of money management and administration; therefore, the more people trust the government, the more willing they are to pay a one-off tax. In the donation-frame condition, on the other hand, trust in NHS is the main determinant of trust in money management (β = 0.34), whereas trust in public institution has a weaker – although significant – relation (β = 0.14). This is in line with the fact that, in the case of monetary donations, people are free to choose the body that will receive the money, including NHS directly, which would become the third party in charge of handling money. Thus, the more people trust the NHS, the more willing they are to make a monetary donation.

Another divergence was found in the relationship between collectivism and the dependent variable. In fact, this relationship is positive and significant in the tax-framed condition (β = 0.14), whereas it is non-significant in the donation-framed condition. Having collectivistic values seems to play a role only in tax payment situations, not in charitable giving.

## Discussion

This study provides novel insights into behavioral responses to pandemics by assessing people’s willingness to financially provide to the NHS during a public health emergency. Instead of using a fictitious situation or simulation experimental design, the present study was able to assess people’s willingness to give such contributions in the midst of the novel coronavirus (COVID-19) emergency situation in Italy.

The aim of this work was twofold. On the one hand, it aimed to investigate the antecedents of citizens’ willingness to financially support the national healthcare system in a situation of public emergency. On the other hand, it aimed to investigate the framing effect of labeling such a financial contribution either as a one-off tax or as a one-off monetary donation.

Results showed that people were more willing to make a financial contribution if it was labeled as a one-off donation rather than as a one-off tax. At a theoretical level, this supports the existence of a “tax aversion” bias, a phenomenon by which people may perceive an additional burden associated with tax payments compared to economically equivalent payments labeled differently (Fennell and Fennell, 2003; McCaffery and Baron, 2006). A further explanation is the distaste for the coercive nature of taxes (as compared with voluntary private giving) and the desire of donors to control or target their donation (Li et al., 2011). Although tax payments and charitable donations are arguably two sides of the same coin – as both are ways to financially provide for the common good – at the affective and psychological levels they are perceived to be very different (Castiglioni et al., 2019b). At the pragmatic level, such evidence can help policymakers and administrators to understand what kind of consensus different policies can achieve among the population during an emergency.

With regard to the antecedents of citizens’ willingness to financially support the national healthcare system, perceived control in terms of trust toward money management appeared to be the most relevant factor. This demonstrates the importance of reassuring people that their financial contributions will be used for the stated purpose (Oosterhof et al., 2009). Although this result equally applied to the tax group and the donation group, different antecedents were found in relation to trust in money management. With regard to tax payment, both general trust in public institutions and general trust in NHS have an impact on trust in money management. Trust in public institutions, however, is more relevant. The importance of trust in the tax compliance domain is not a novelty, as also suggested by the “slippery slope framework” (Kirchler et al., 2008; Gangl et al., 2015). According to the “slippery slope framework,” when tax authorities are trusted, people are more willing to be voluntary tax compliant. In addition, tax compliance increases when taxpayers are aware of a direct link between their tax payments and the provision of a desirable public good (Alm et al., 1992). The present study underlines the impact and the importance of the proper management of tax money, not only in a situation of ordinary administration but also in a situation of emergency. In addition, it expands the role and importance of trust not only toward tax authorities but also toward public institutions at large.

In the charitable giving condition, on the other hand, only general trust in NHS appears to be an antecedent of trust in money management. This can be explained by the fact that, in the case of monetary donations, people can directly donate to the NHS, with no mediation of other public institutions. Therefore, trusting that the NHS will properly handle their money is the main determinant.

According to the model we tested, perceived trust was even more important than subjective norms, which appeared to be significant but to a lesser extent. In particular, the “accessibility” component of orientation toward the common good, but not “personal gain,” predicted behavioral intention. In the midst of a public health emergency, making sure that all the citizens can have access to adequate health assistance appears to be more relevant in predicting the intention to give a financial contribution than pursuing personal interests. Collectivism appeared to be a significant antecedent as well. However, when distinguishing between the tax group and the donation group, it appeared to be significant only in the tax-framed condition. Donation literature on the “warm glow” feeling can explain the lack of impact of collectivism in the donation-framed condition. According to the “warm glow” theory, donors gain utility not only from increasing public goods but also from the act of giving itself (impure altruism; Andreoni, 1990). That is, people may have pleasurable psychological experiences upon donating money, and such feelings may be more important to the donors than actually increasing the collective welfare (Butera and Horn, 2020).

Finally, an unexpected result was the lack of a significant effect of pandemic attitudes and perceived risk on the intention to make a financial contribution. A possible explanation for the lack of a significant effect may be related to the specificity of the dependent variable in this study (i.e., intention to give a financial contribution to the NHS). Although people’s risk perception of the pandemic is one of the key factors contributing to an increase in public participation in adopting preventive measures (Rogers, 1975; Prati et al., 2011) and vaccination acceptance (Van der Weerd et al., 2011), this might not apply to making a financial contribution to the common good. A possible explanation for the lack of significant effects of perceived risk on the intention to make a financial contribution to the NHS is related to the specific timing with which we collected the data. As mentioned above, data were gathered during the peak of the coronavirus outbreak in Italy, and most respondents lived in Lombardy, the most affected region in Italy. Given the “objectivity” of the seriousness and severity of the pandemic emergency at the time people took part in the study, the role played by their “subjective” perception may have been mitigated. Further research on the topic may help gain a better understanding of such results.

To summarize, at a pragmatic level, results suggest that a social communication aimed at collecting financial support to face a health emergency situation should emphasize how money will be handled and managed, rather than focusing on the potential health risks associated with the pandemic spread. Moreover, social marketing campaigns aimed at increasing tax compliance need to reassure people regarding how public institutions will use the money, as well as foster important social values related to the accessibility of public goods and collectivism. Meanwhile, in social marketing campaigns aimed at collecting money from private donations, it is important to support trust toward the NHS and emphasize the accessibility dimension of the common good.

Our study had several limitations that should be noted. First, results were self-reported. Measurement errors and social desirability bias may exist despite the fact that the questionnaire was anonymous. A further limitation of this study is that we assessed behavioral intentions rather than objectively measured behavior. Although being very important and well-validated proxy for behavior, it is known that behavioral intentions can only predict a moderate amount (30–42%) of the variance in actual behavior across a wide range of contexts (Armitage and Conner, 2000; Webb and Sheeran, 2006; Cooke and French, 2008). Finally, results cannot be generalized in a probabilistic way to the whole Italian population, as a non-probabilistic sampling technique was adopted. However, the present study did not aim to perform a probabilistic generalization, but rather an analytic generalization, which relies on the design of the research to make casual claims in relation to a model/theory. In that regard, the present study needs to be considered as a first exploration on the topic. Further studies taking into account other sources of variability, such as different Italian regions, different times, different countries (e.g., high-tax compliance countries vs. low-tax compliance countries), and with different kinds of emergencies (i.e., not health-related) may provide further insights.

The study has several strengths too. First, it was conducted during the threat of an actual new influenza pandemic, during its contagion peak. It therefore provides important insights into the reactions of the public, in addition to the number of laboratory and hypothetical studies on people’s reactions to pandemics. Second, our model proved a very good fit to the data and gave important insights into understanding the antecedents of the financial provision for the common good. It also stressed differences and specificities of two different domains: tax payment and charitable giving. Third, in addition to the theoretical results, it also offers operational suggestions for policy-makers and administrators in facing a health-related emergency.

## Conclusion

Ensuring fiscal sustainability has become a priority challenge for many countries, including Italy. If this is true in times of normal economic growth, it becomes even more relevant in times of crisis. The public health emergency related to the novel coronavirus (COVID-19) has further weakened many countries’ public finances, thus making it of paramount importance to understand how to promote citizens’ contribution to the common good. From a financial point of view, this includes paying taxes and giving money to charities. This specific sustainability goal fits into the larger picture of the 2030 Agenda for Sustainable Development, which includes a set of 17 Sustainable Development Goals (SDGs), as it was established at the 70th Session of the UN General Assembly in 2015.

This study’s outcomes are important for governmental crisis communication. It is suggested that, rather than focusing on potential risks and fear-arousal messages, social communication aimed at collecting financial resources to support the NHS should provide information on how the money will be handled, in order to increase trust in money management and increase a sense of perceived control.

## Data Availability Statement

The raw data supporting the conclusion of this article will be made available by the authors, without undue reservation.

## Ethics Statement

Ethical review and approval was not required for the study on human participants in accordance with the local legislation and institutional requirements. The patients/participants provided their written informed consent to participate in this study.

## Author Contributions

Both authors contributed to the conception and design of the study, contributed to manuscript revision, read and approved the submitted version. CC analyzed the data and wrote the first draft of the manuscript.

## Conflict of Interest

The authors declare that the research was conducted in the absence of any commercial or financial relationships that could be construed as a potential conflict of interest.

## Footnotes

1 Data retrieved from the official Ministero della Salute website, http://www.salute.gov.it/, on July 16th, 2020.

2 https://ec.europa.eu/eurostat/tgm/table.do?tab=tableandinit=1andlanguage=enandpcode=teina225andplugin=1

3 https://www.bva-doxa.com/en/feelings-habits-and-attitudes-italians-in-covid-19-times/

## References

[B1] Ahmad A. Mueller C. Tsamakis K. (2020). Covid-19 pandemic: a public and global mental health opportunity for social transformation?. *BMJ* 369:m1383 10.1136/bmj.m1383 32253252

[B2] Ajzen I. (1991). The theory of planned behavior. *Organ. Behav. Hum. Dec. Process.* 50 179–211.

[B3] Alm J. Jackson B. R. McKee M. (1992). Estimating the determinants of taxpayer compliance with experimental data. *Natl. Tax J.* 45 107–114.

[B4] Andreoni J. (1990). Impure altruism and donations to public goods: a theory of warm-glow giving. *Econ. J.* 100 464–477. 10.2307/2234133

[B5] Arbuckle J. L. (2003). *Amos 5.0.* Chicago, IL: SPSS.

[B6] Armitage C. J. Conner M. (2000). Social cognition models and health behaviour: a structured review. *Psychol. Health* 15 173–189. 10.1080/08870440008400299

[B7] Barr M. Raphael B. Taylor M. Stevens G. Jorm L. Giffin M. (2008). Pandemic influenza in Australia: using telephone surveys to measure perceptions of threat and willingness to comply. *BMC Infect. Dis.* 8:117. 10.1186/1471-2334-8-117 18793441 PMC2556339

[B8] Bootsma M. C. Ferguson N. M. (2007). The effect of public health measures on the 1918 influenza pandemic in US cities. *Proc. Natl. Acad. Sci. U S A* 104, 7588–7593. 10.1073/pnas.0611071104 17416677 PMC1849868

[B9] Brug J. Aro A. R. Richardus J. H. (2009). Risk perceptions and behaviour: towards pandemic control of emerging infectious diseases. *Int. J. Behav. Med.* 16:3. 10.1007/s12529-008-9000-x 19127440 PMC7090788

[B10] Butera L. Horn J. (2020). “Give less but give smart”: experimental evidence on the effects of public information about quality on giving. *J. Econ. Behav. Organ.* 171 59–76. 10.1016/j.jebo.2020.01.011

[B11] CAF Charities Aid Foundation (2019). *CAF World Giving Index*, 10th Edn Available online at: https://www.cafonline.org/about-us/publications/2019-publications/caf-world-giving-index-10th-edition (accessed November 1, 2020).

[B12] Cartabellotta N. Cottafava E. Luceri R. Mosti M. (2019). *4° Rapporto sulla sostenibilità del Servizio Sanitario Nazionale.* Available online at: https://www.rapportogimbe.it/4_Rapporto_GIMBE.pdf (accessed November 1, 2020).

[B13] Castiglioni C. Lozza E. Bonanomi A. (2019a). The common good provision scale (CGP): a tool for assessing people’s orientation towards economic and social sustainability. *Sustainability* 11:370 10.3390/su11020370

[B14] Castiglioni C. Lozza E. Bosio A. C. (2018). Lay people representations on the common good and its financial provision. *SAGE Open* 8:2158244018807247 10.1177/2158244018807247

[B15] Castiglioni C. Lozza E. van Dijk E. van Dijk W. W. (2019b). Two sides of the same coin? An investigation of the effects of frames on tax compliance and charitable giving. *Palgrave Commun.* 5 1–10. 10.1057/s41599-019-0247-4

[B16] Cooke R. French D. P. (2008). How well do the theory of reasoned action and theory of planned behaviour predict intentions and attendance at screening programmes? A meta-analysis. *Psychol. Health* 23 745–765. 10.1080/08870440701544437 25160879

[B17] Di Fabio A. Rosen M. A. (2018). Opening the black box of psychological processes in the science of sustainable development: a new frontier. *Eur. J. Sustain. Dev. Res.* 2:47 10.20897/ejosdr/3933

[B18] Fennell C. C. Fennell L. A. (2003). Fear and greed in tax policy: a qualitative research Agenda. *Washington Univ. J. Law Policy* 13:75.

[B19] Ferrari L. Randisi S. (2011). *Psicologia fiscale. Illusioni e Decisioni dei Contribuenti.* Milano: Raffaello Cortina Editore.

[B20] Gangl K. Hofmann E. Kirchler E. (2015). Tax authorities’ interaction with taxpayers: a conception of compliance in social dilemmas by power and trust. *New Ideas Psychol.* 37 13–23. 10.1016/j.newideapsych.2014.12.001 25859096 PMC4381354

[B21] Giovannini E. (2011). *Economia Non Osservata e Flussi Finanziari. Rapporto Finale.* Roma: Ministero dell’Economia e delle Finanze.

[B22] Goodwin R. Haque S. Neto F. Myers L. B. (2009). Initial psychological responses to Influenza A, H1N1 (“Swine flu”). *BMC Infect. Dis.* 9:166. 10.1186/1471-2334-9-166 19807908 PMC2765446

[B23] Jones K. (2017). Government or charity? Preferences for welfare provision by ethnicity. *J. Behav. Exp. Econ.* 66 72–77. 10.1016/j.socec.2016.04.011

[B24] Kirchler E. Hoelzl E. Wahl I. (2008). Enforced versus voluntary tax compliance: the “slippery slope” framework. *J. Econ. Psychol.* 29 210–225. 10.1016/j.joep.2007.05.004

[B25] Lau J. T. Kim J. H. Tsui H. Y. Griffiths S. (2007). Anticipated and current preventive behaviors in response to an anticipated human-to-human H5N1 epidemic in the Hong Kong Chinese general population. *BMC Infect. Dis.* 7:18. 10.1186/1471-2334-7-18 17359545 PMC1845150

[B26] Li S. X. Eckel C. C. Grossman P. J. Brown T. L. (2011). Giving to government: voluntary taxation in the lab. *J. Public Econ.* 95 1190–1201. 10.1016/j.jpubeco.2011.03.005

[B27] McCaffery E. J. Baron J. (2006). Thinking about tax. *Psychol. Public Policy Law* 12 106–135. 10.1037/1076-8971.12.1.106

[B28] Nicoli F. Gasparetto A. (2020). Italy in a time of emergency and scarce resources: the need for embedding ethical reflection in social and clinical settings. *J. Clin. Ethics* 31 92–94. 32213700

[B29] Oosterhof L. Heuvelman A. Peters O. (2009). Donation to disaster relief campaigns: Underlying social cognitive factors exposed. *Eval. Program. Plann.* 32, 148–157. 10.1016/j.evalprogplan.2008.10.006 19081135

[B30] Poletti P. Ajelli M. Merler S. (2011). The effect of risk perception on the 2009 H1N1 pandemic influenza dynamics. *PLoS One* 6:e0016460. 10.1371/journal.pone.0016460 21326878 PMC3034726

[B31] Prati G. Pietrantoni L. Zani B. (2011). A social-cognitive model of pandemic influenza H1N1 risk perception and recommended behaviors in Italy. *Risk Anal.* 31 645–656. 10.1111/j.1539-6924.2010.01529.x 21077927

[B32] Rogers R. W. (1975). A protection motivation theory of fear appeals and attitude change1. *J. Psychol.* 91 93–114. 10.1080/00223980.1975.9915803 28136248

[B33] Rubin G. J. Amlôt R. Page L. Wessely S. (2009). Public perceptions, anxiety, and behaviour change in relation to the swine flu outbreak: cross sectional telephone survey. *BMJ* 339:b2651. 10.1136/bmj.b2651 19574308 PMC2714687

[B34] Sadique M. Z. Edmunds W. J. Smith R. D. Meerding W. J. de Zwart O. Brug J. (2007). Precautionary behavior in response to perceived threat of pandemic influenza. *Emerg. Infect. Dis.* 13 1307–1313. 10.3201/eid1309.070372 18252100 PMC2857294

[B35] Santoro A. (2010). *L’evasione Fiscale.* Bologna, Italy: Il Mulino.

[B36] Schiefer D. van der Noll J. (2017). The essentials of social cohesion: a literature review. *Soc. Indic. Res.* 132 579–603. 10.1007/s11205-016-1314-5

[B37] Schneider F. (2015). Size and development of the shadow economy of 31 European and 5 other OECD countries from 2003 to 2014: different developments? *J. Self Govern. Manag. Econ.* 3 7–29.

[B38] Selle P. (1993). Voluntary organisations and the welfare state: the case of Norway. *Voluntas Int. J. Volunt. Nonprofit Organ.* 4 1–15. 10.1007/BF01398382

[B39] Slavov S. N. (2014). Public versus private provision of public goods. *J. Public Econ. Theory* 16 222–258. 10.1111/jpet.12058

[B40] Smith R. D. Keogh-Brown M. R. Barnett T. Tait J. (2009). The economy-wide impact of pandemic influenza on the UK: a computable general equilibrium modelling experiment. *BMJ* 339:b4571. 10.1136/bmj.b4571 19926697 PMC2779854

[B41] Sugden R. (1984). Reciprocity: the supply of public goods through voluntary contributions. *Econ. J.* 94 772–787. 10.2307/2232294

[B42] Triandis H. C. Gelfand M. J. (1998). Converging measurement of horizontal and vertical individualism and collectivism. *J. Pers. Soc. Psychol.* 74, 118–128. 10.1037/0022-3514.74.1.118

[B43] United Nations (2018). *About the Sustainable Development Goals.* Available online at: https://www.un.org/sustainabledevelopment/sustainable-development-goals (accessed November 1, 2020).

[B44] Van der Weerd W. Timmermans D. R. Beaujean D. J. Oudhoff J. Van Steenbergen J. E. (2011). Monitoring the level of government trust, risk perception and intention of the general public to adopt protective measures during the influenza A (H1N1) pandemic in the Netherlands. *BMC Public Health* 11:575. 10.1186/1471-2458-11-575 21771296 PMC3152536

[B45] Webb T. L. Sheeran P. (2006). Does changing behavioral intentions engender behavior change? A meta-analysis of the experimental evidence. *Psychol. Bull.* 132:249. 10.1037/0033-2909.132.2.249 16536643

[B46] Xu Z. Shi L. Wang Y. Zhang J. Huang L. Zhang C. (2020). Pathological findings of COVID-19 associated with acute respiratory distress syndrome. *Lancet Resp. Med.* 8 420–422. 10.1016/S2213-2600(20)30076-X PMC7164771 32085846

[B47] Yoo B. Donthu N. Lenartowicz T. (2011). Measuring Hofstede’s five dimensions of cultural values at the individual level: development and validation of CVSCALE. *J. Int. Consumer Mark.* 23 193–210. 10.1080/08961530.2011.578059

